# Performance Evaluation of Real-Time Precise Point Positioning with Both BDS-3 and BDS-2 Observations

**DOI:** 10.3390/s20216027

**Published:** 2020-10-23

**Authors:** Lin Pan, Xuanping Li, Wenkun Yu, Wujiao Dai, Cuilin Kuang, Jun Chen, Fade Chen, Pengfei Xia

**Affiliations:** 1 School of Geosciences and Info-Physics, Central South University, Changsha 410083, China; linpan@csu.edu.cn (L.P.); xpli1997@csu.edu.cn (X.L.); wjdai@csu.edu.cn (W.D.); kuangcuilin@csu.edu.cn (C.K.); 2 Guangxi Key Laboratory of Spatial Information and Geomatics, Guilin 541004, China; 3 Hunan Institute of Surveying and Mapping Technology, Changsha 410007, China; 4 School of Geodesy and Geomatics, Wuhan University, 129 Luoyu Road, Wuhan 430079, China; cj_snake@whu.edu.cn (J.C.); fadechen@whu.edu.cn (F.C.); 5 GNSS Research Center, Wuhan University, 129 Luoyu Road, Wuhan 430079, China; pfxia130@whu.edu.cn

**Keywords:** BDS-3, BDS-2, real-time, precise point positioning

## Abstract

For time-critical precise applications, one popular technology is the real-time precise point positioning (PPP). In recent years, there has been a rapid development in the BeiDou Navigation Satellite System (BDS), and the constellation of global BDS (BDS-3) has been fully deployed. In addition to the regional BDS (BDS-2) constellation, the real-time stream CLK93 has started to support the BDS-3 constellation, indicating that the real-time PPP processing involving BDS-3 observations is feasible. In this study, the global positioning performance of real-time PPP with BDS-3/BDS-2 observations is initially evaluated using the datasets from 147 stations. In the east, north and upward directions, positioning accuracy of 1.8, 1.2 and 2.5 cm in the static mode, and of 6.7, 5.1 and 10.4 cm in the kinematic mode can be achieved for the BDS-3/BDS-2 real-time PPP, respectively, while the corresponding convergence time with a threshold of 10 cm is 32.9, 23.7 and 32.8 min, and 66.9, 42.9 and 69.1 min in the two modes in the three directions, respectively. To complete this, the availability of BDS-3/BDS-2 constellations, the quality of BDS-3/BDS-2 real-time precise satellite products, and the BDS-3/BDS-2 post-processed PPP solutions are also analyzed. For comparison, the results for the GPS are also presented.

## 1. Introduction

With a view to the need for social and economic development as well as national security, China independently constructs and operates the BeiDou Navigation Satellite System (BDS), which is one of the Global Navigation Satellite Systems (GNSSs). BDS is an important national temporal–spatial infrastructure that is committed to providing all-weather and all-time positioning, navigation and timing (PNT) service with high accuracies for users all over the world. For the development and construction of BDS, great importance is attached by China. China has been exploring a path to develop a navigation satellite system suitable for its national conditions since the 1980s. A development strategy characterized by three steps was gradually formulated. The BeiDou navigation demonstration system (BDS-1) was built in the first step. In 1994, China started BDS-1 construction. In 2000, China launched two satellites in geostationary orbit (GEO), and then put BDS-1 into use. For the users in China, BDS-1 could provide timing, positioning, a wide-area differential as well as short-message communication service with the use of an active positioning scheme. For the purpose of the further enhancement of BDS-1 performance, China launched the third GEO satellite in 2003. The regional BDS (BDS-2) was built in the second step. In 2004, China started BDS-2 construction. Fourteen BDS-2 satellites were launched by the end of 2012, including five GEO, five inclined geosynchronous orbit (IGSO) and four medium earth orbit (MEO) satellites. BDS-2 and BDS-1 adopted the compatible technical scheme, but for users in the Asia-Pacific region, the passive positioning scheme was further included to provide PNT, velocity measurement and short message communication service. The global BDS (BDS-3) was built in the third step. In 2009, China started BDS-3 construction. By 2020, BDS-3 will be completed in an all-round way (i.e., with full operational capability), with 30 launched satellites (24 MEO/3 IGSO/3 GEO). Two technical schemes, namely passive service and active service, were inherited for BDS-3. For global users, the PNT, international search and rescue, and global short-message communication service can be provided by BDS-3, while for users in China and adjacent regions, the regional short message communication, precise point positioning (PPP), ground-based augmentation, and satellite-based augmentation service can be further offered. China completed the basic constellation deployment of BDS-3 on 27 December 2018, and BDS-3 started to provide global services. The core constellation deployment of BDS-3 was completed on 16 December 2019 with a successful launch of the 24th BDS-3 MEO satellite, and the performance of global services was further improved. On 23 June 2020, the last global networking satellite of BDS-3 was successfully launched, and the full constellation deployment of BDS-3 was officially finished.

BDS-2 data processing has drawn a lot of attention from the GNSS community in the past several years [1,2,3,4,5]. With the availability of BDS-3 real data, many researchers focused on the various aspects of BDS-3, including broadcast ephemeris [6], satellite signals [7], global broadcast ionospheric delay correction model [8,9], satellite-induced code bias [10], time group delay (TGD) [11], orbit determination [12,13,14], differential code bias (DCB) [15,16], orbit and clock analysis [17], and time and frequency transfer [18]. In actuality, for a navigation satellite system, positioning is one of the core functions and basic services. GNSS-based PPP is an absolute positioning technology, which employs the GNSS observations collected by a single receiver as well as the precise products of satellite clocks and orbits from international GNSS service (IGS) [19,20]. In addition to the high position accuracies (decimeter-to-centimeter-level kinematic position accuracies and centimeter-to-millimeter-level static position accuracies), the PPP technology is characterized by the absent reference station, no limitation of operating distance, simple data processing, low cost, and flexibility [21,22]. Thus, in the past two decades, PPP technology has been a research focus. Several studies also concentrated on PPP processing involving BDS-3 measurements. Zhang et al. [23] investigated the contribution of BDS-3 observations to the BDS-2 PPP using the datasets from three stations capable of tracking the full BDS-3 and BDS-2 constellations. Both the position accuracies and convergence speed of BDS-3/BDS-2 kinematic PPP were improved compared with the BDS-2 kinematic PPP. Based on the datasets from ten stations, Shi et al. [24] also validated the superior performance of BDS-3/BDS-2 PPP in comparison to the BDS-2 PPP. In addition, continuous positioning became feasible for the stations located in Germany and North/South America after introducing the BDS-3 measurements into BDS-2 PPP processing. Zhang et al. [25] proposed a BDS-3/BDS-2 triple-frequency uncombined PPP model, in which the stochastic plus deterministic ionosphere model was used to constrain the estimated ionosphere delays. To indicate the potential of BDS-3 and BDS-2 integration, they also compared the BDS-3/BDS-2 PPP solutions with the standalone BDS-2 PPP solutions. Zhang et al. [26] assessed the performance of kinematic and static BDS-3 PPP using the datasets at two stations. After a convergence time of four hours, the horizontal and vertical position accuracies reached the centimeter level and decimeter level, respectively.

Because of the latency of precise products of satellite clocks and orbits, the previous studies about BDS-3 PPP were confined to a post-processed mode. There has been a rapid growth in demand for real-time precise applications for the GNSS community in recent years [27,28,29,30,31], such as precision agriculture and tsunami warning system. The applications of BDS-3 post-processed PPP will be severely limited in these time-critical applications. The real-time stream CLK93 transmitted by Centre National d’Etudes Spatiales (CNES) starts to support the BDS-3 constellation, in addition to the BDS-2 constellation. Thus, it is feasible to investigate real-time PPP performance with BDS-3 observables. To fully take advantage of the BDS potential, the real-time PPP performance with BDS-3/BDS-2 observations in kinematic and static modes is evaluated in this study. To complete this, the availability of BDS-3/BDS-2 constellations (including the PNT service rate, the Position Dilution of Precision (PDOP), and the number of visible satellites), and the qualities of BDS-3/BDS-2 real-time precise products are also analyzed. For the purpose of comparison, the post-processed BDS-3/BDS-2 PPP solutions as well as the corresponding GPS results (including the real-time PPP solutions, post-processed PPP solutions, availability of satellite constellation, and qualities of real-time precise products) are also presented. This paper starts with the real-time BDS-3/BDS-2 PPP model. Next, we describe the current space segment of BDS-3/BDS-2 constellations. Subsequently, the results are provided and discussed. Finally, the main conclusion is summarized.

## 2. Real-Time BDS-3/BDS-2 PPP Model

The ionospheric-free (IF) combination is adopted in the real-time BDS-3/BDS-2 PPP model, for the purpose of eliminating first-order effects of ionospheric delay. The overlap signals only include B3 and B1 for BDS-2 and BDS-3 satellites. The B1/B3 dual-frequency IF combined phase and code observables are exhibited as follows:

(1) $$\{\begin{array}{l} P_{IF}=a_{13,1}\times P_{1}+a_{13,3}\times P_{3}=\rho +cdt_{r}-cdt+T+d_{r,IF}+d_{IF}+\varepsilon_{P_{IF}}\\ \Phi_{IF}=a_{13,1}\times \Phi_{1}+a_{13,3}\times \Phi_{3}=\rho +cdt_{r}-cdt+T+N_{IF}+b_{r,IF}+b_{IF}+\varepsilon_{\Phi_{IF}} \end{array}$$

with

(2) $$\{\begin{array}{l} N_{IF}=a_{13,1}\times N_{1}+a_{13,3}\times N_{3}\\ d_{r,IF}=a_{13,1}\times d_{r,1}+a_{13,3}\times d_{r,3}\\ d_{IF}=a_{13,1}\times d_{1}+a_{13,3}\times d_{3}\\ b_{r,IF}=a_{13,1}\times b_{r,1}+a_{13,3}\times b_{r,3}\\ b_{IF}=a_{13,1}\times b_{1}+a_{13,3}\times b_{3} \end{array}$$

(3) $$\{\begin{array}{l} a_{13,1}=\frac{f_{1}^{2}}{f_{1}^{2}-f_{3}^{2}}\\ a_{13,3}=-\frac{f_{3}^{2}}{f_{1}^{2}-f_{3}^{2}} \end{array}$$

where $P_{i}\;(i=\;1,\;3)$ denotes the code observations on B1 or B3 frequency, $\Phi_{i}$ is the corresponding phase observations, $a_{13,i}$ is the dual-frequency IF combination coefficient, $f_{i}$ denotes the carrier frequency, $d_{i}$ and $d_{r,i}$ are the code hardware delays at the satellite and receiver, respectively, $N_{i}$ is the phase ambiguity, $b_{i}$ and $b_{r,i}$ denote the satellite- and receiver-specific phase hardware delays, respectively, $P_{IF}$, $\Phi_{IF}$, $N_{IF}$, $d_{r,IF}$, $d_{IF}$, $b_{r,IF}$ and $b_{IF}$ are the corresponding terms for the dual-frequency IF combination, $\varepsilon_{P_{IF}}$ and $\varepsilon_{\Phi_{IF}}$ are the code and phase observation noises, respectively, T is the slant tropospheric delay, $cdt_{r}$ and cdt are the physical clock errors of the receiver and the satellite, respectively, and ρ denotes the geometric distances. For a period of time, all the hardware delay terms are considered to be stable [32].

The corrections of satellite clocks in the real-time scenario, which are contained in the real-time stream CLK93 provided by CNES, are computed with the use of B1/B3 dual-frequency IF combination. Since the hardware delay terms are ignored when generating the real-time stream CLK93, the derived corrections of satellite clocks in the real-time situation include a B1/B3 dual-frequency IF combination of code hardware delays at the satellite, besides the physical clock errors. With the use of real-time precise products, the satellite clocks and orbits are fixed. We estimate the wet component of tropospheric delay from the observations, and employ an a priori model to correct the dry component. After grouping the parameters with similar characteristics, the linearized observation model of real-time BDS-3/BDS-2 PPP reads:

(4) $$\{\begin{array}{l} p_{IF}=\mu \times X+c\hat{d}t_{r}+m\times Z+\varepsilon_{p_{IF}}\\ \phi_{IF}=\mu \times X+c\hat{d}t_{r}+m\times Z+{\hat{N}}_{IF}+\varepsilon_{\phi_{IF}} \end{array}$$

with

(5) $$\{\begin{array}{l} c\hat{d}t_{r}=cdt_{r}+d_{r,IF}\\ {\hat{N}}_{IF}=N_{IF}+b_{r,IF}+b_{IF}-d_{r,IF}-d_{IF} \end{array}$$

where $\Phi_{IF}$ and $p_{IF}$ denote the OMC (observed-minus-computed) phase and code observables of IF combination, respectively, μ denotes the unit vector in line-of-sight direction, X denotes the three-dimensional (3D) receiver positions, $c\hat{d}t_{r}$ denotes the receiver clock estimates including code hardware delays at the receiver, ${\hat{N}}_{IF}$ is the phase ambiguity estimates grouped with phase and code hardware delays at both satellite and receiver ends, Z is the tropospheric zenith wet delay (ZWD), m is the wet mapping function, and $\varepsilon_{p_{IF}}$ and $\varepsilon_{\varphi_{IF}}$ are the noise terms of $p_{IF}$ and $\Phi_{IF}$, respectively. Although we do not display the relativistic effect, phase windup effect, station displacement, phase center variation (PCV) and phase center offset (PCO) in Equation (4), the corresponding models or products should be used to mitigate them [19,33].

The estimated parameters cover ZWD, phase ambiguity, receiver clocks, and receiver positions, that is:

(6) $$S={\left[X,c\hat{d}t_{r},Z,{\hat{N}}_{IF}\right]}^{T}$$

where S denotes the vector of estimates. In this study, for the parameter estimation, we employ a Kalman filter. Both static receiver positions and phase ambiguity parameters are estimated as constants, while white noise processes are adopted for kinematic receiver positions and receiver clock parameters. As for the ZWD, it is modeled as random-walk processes. In this study, the open-source software RTKLIB [34] is used, but with many improvements, such as the stochastic model.

As the observations at low elevations are vulnerable to the measurement noises and multipath errors, the elevation-dependent weighting schemes are employed for real-time BDS-3/BDS-2 PPP regarding the stochastic model, that is:

(7) $$\sigma^{2}\left(ele\right)=w^{2}\cdot \left(a^{2}+\frac{b^{2}}{{\left(\sin ele\right)}^{2}}\right)$$

where $\sigma^{2}\left(ele\right)$ is the variance of code or phase observations with an elevation angle ele, and a and b are two constants, both of which can be taken as 0.003 and 0.3 m for the phase and code measurements, respectively. w is the weight factor to consider the different effects of the accuracy of the precise clock and orbit corrections of satellites in the real-time situation among different satellite constellations. Following Kazmierski et al. [35], a good positioning performance can be achieved when the weight factor is proportional to the numerical value of the signal-in-space ranging error (SISRE). The weight factor is roughly set to 1.0, 1.0, 1.5 and 6.0 for BDS-2 MEO, BDS-3 MEO, BDS-2 IGSO and BDS-2 GEO satellites, respectively, according to the results of accuracy evaluation for the real-time precise products in this study.

## 3. Current Space Segment of BDS-3/BDS-2 Constellations

**Figure 1:**
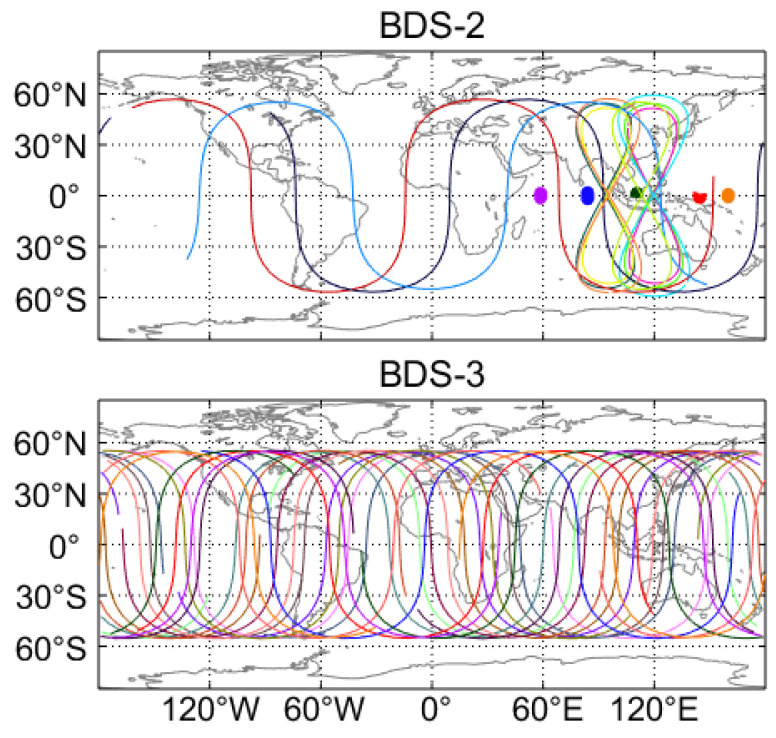
Ground track of BDS-2 and BDS-3 satellites on day of the year (DOY) 140, 2020. Different colors in the figure refer to different satellites.

As of September 2020, the in-orbit BDS satellites include 24 MEO, 3 IGSO and 3 GEO satellites for BDS-3, and 3 MEO, 7 IGSO and 6 GEO satellites for BDS-2. Table 1 details the current space segment of BDS-3 and BDS-2. In addition to the BDS-3/BDS-2 satellites, there are also five in-orbit validation satellites of BDS-3, namely BDS-3S satellites. The PRN is not clear for one BDS-3S MEO satellite which suffers from a failure of the transmitting antenna. So far, there is no receiver tracking of this satellite. The BDS satellites with a PRN in the parenthesis in Table 1 cannot be supported by the real-time stream CLK93, including one BDS-2 GEO satellite (i.e., C18), all BDS-3S satellites, and all BDS-3 satellites except for the MEO satellites with a PRN smaller than C38. Currently, the real-time stream CLK93 can offer the precise corrections of 18 BDS-3 MEO, 3 BDS-2 MEO, 7 BDS-2 IGSO and 5 BDS-2 GEO satellites. The orbital altitude is 35,786 km for the GEO and IGSO satellites, whereas the MEO satellites with an altitude of 21,528 km operate in nearly circular orbits. For the GEO satellites, the non-zero inclination falls between 0.7° and 1.7°, while both MEO and IGSO satellites are inclined at 55°. As for the orbit period and orbital repeat period, they are 12 h 53 min and approximately a week for MEO satellites, respectively, but both of them are 23 h 56 min for non-MEO satellites. The ground track for BDS-3/BDS-2 satellites supported by real-time precise products on the day of the year (DOY) 140 of 2020 is illustrated in Figure 1. Except for the Arctic and Antarctica regions, most regions of the world are covered by the ground track of MEO satellites of BDS-3 and BDS-2. Two figure-of-eight loops with a respective ascending node longitude of 118° E and 95° E can be identified with respect to the ground track of BDS-2 IGSO satellites. For the five BDS-2 GEO satellites (i.e., C01–C05), the ground track is fixed over the equator at 140° E, 84° E, 110.5° E, 160° E and 58.75° E longitude, respectively. Thus, in the performance enhancement of BDS PPP, both the IGSO and GEO satellites have potential for users in the Asia-Pacific regions. As for the GPS system, the corresponding information is detailed in Pan et al. [36].

## 4. Results and Discussion

### 4.1. Experimental Datasets

**Figure 2:**
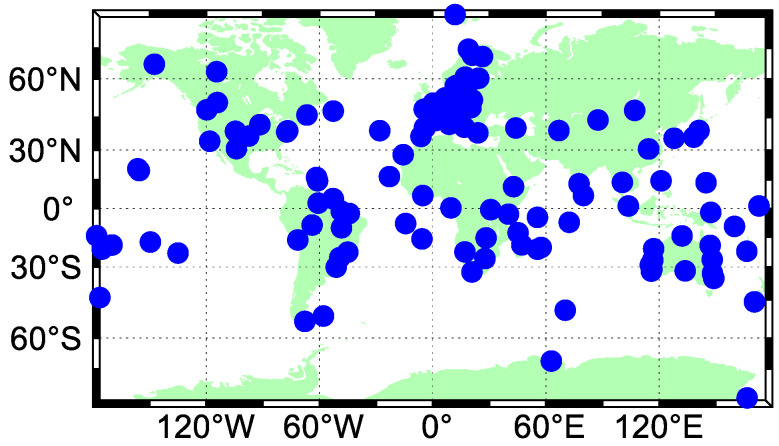
Geographical distribution of stations used in the analysis.

The datasets from a total of 147 Multi-GNSS Experiment (MGEX) stations, at which BDS-3 and BDS-2 signals can be both tracked, are used for the performance evaluation of BDS-3/BDS-2 real-time PPP. The geographical distribution of stations used in our analysis is shown in Figure 2. The cut-off elevation is set to 7°. The sampling interval of the BDS-3/BDS-2 observations is 30 s. It should be noted that the positioning performance of real-time PPP can only be substantially assessed with the use of an observation sampling rate of 30 s. When the sampling interval of observations is reduced, the convergence time of real-time PPP is expected to be shortened [37]. The real-time stream CLK93 released by CNES is employed to acquire the precise corrections for the satellite clocks and orbits in the real-time scenario, on the basis of broadcast ephemeris. The data rate of the CLK93 stream is 5 s. The analysis period covers 70 days from DOY 124 to 193 of 2020 for the assessment of real-time precise product quality, while a time span of a week from DOY 140 to 146 of 2020 is employed for the availability analysis of satellite constellations and the evaluation of real-time PPP performance.

### 4.2. Availability of BDS-3/BDS-2 Constellations

We evaluate the global availability of BDS-3 and BDS-2 combined constellation in this section in terms of the PNT service rate, PDOP, and the number of visible satellites. We also present the results of the GPS constellation, for comparison. Our evaluation is based on the final precise satellite orbit products from Wuhan University (WHU) spanning one week (DOY 140–146 of 2020), which is approximately one repeat cycle for all types of BDS constellations (i.e., MEO/IGSO/GEO) and seven repeat cycles of GPS constellation. The real-time precise products from CNES are not employed due to their frequent interruption. It is important to note that the tracking satellites are identical for the CNES and WHU precise products in terms of BDS-3/BDS-2 and GPS. Thus, only those satellites supported by the CLK93 stream are included in the availability analysis. In this section, we just intend to carry out an optimal availability evaluation for the BDS-3/BDS-2 real-time PNT. The global regions are divided into 72 × 72 grids every 2.5° in latitude and 5° in longitude, respectively. In each grid, it is considered that there is a virtual ground tracking station at the center. Following Yang et al. [38], the altitude of the virtual stations is set to 25 m. The sampling interval is 15 min.

**Figure 3:**
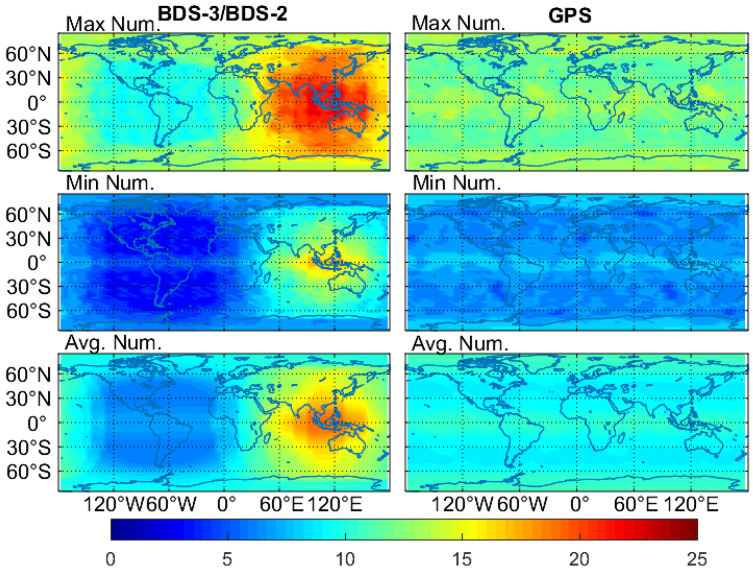
Global maps of the maximum, minimum and average number of visible satellites of GPS and BDS-3/BDS-2 on DOY 140–146 of 2020 (note that a visible satellite has an elevation angle higher than 7°).

The global maps for the number of visible satellites are shown in Figure 3. The maximum value for the maximum number and a minimum number of BDS-3/BDS-2 visible satellites is 23 and 17 in Asia-Pacific regions, respectively, and the corresponding minimum value is 9 and 3 in the Americas, respectively, as can be seen in Figure 3. The average number of BDS-3/BDS-2 visible satellites ranges from 6 to 19 on a global scale. The visible GPS satellites show a less regional difference compared to that of BDS-3/BDS-2, and for the maximum, minimum and average number of GPS satellites, the global varying range is 11–14, 5–9 and 8.8–10.7, respectively. The average satellite number of BDS-3/BDS-2 is 13–19 in the BDS-2 service regions (55° S–55° N latitude and 70° E–150° E longitude), while the corresponding satellite number is 6–9.2 in the 0°–150° W longitude and 60° S–60° N latitude areas. Regarding these two geographical areas, we can observe 6.8 more BDS-3/BDS-2 satellites on average than GPS in the BDS-2 service area, but 2.6 less in the other one.

**Figure 4:**
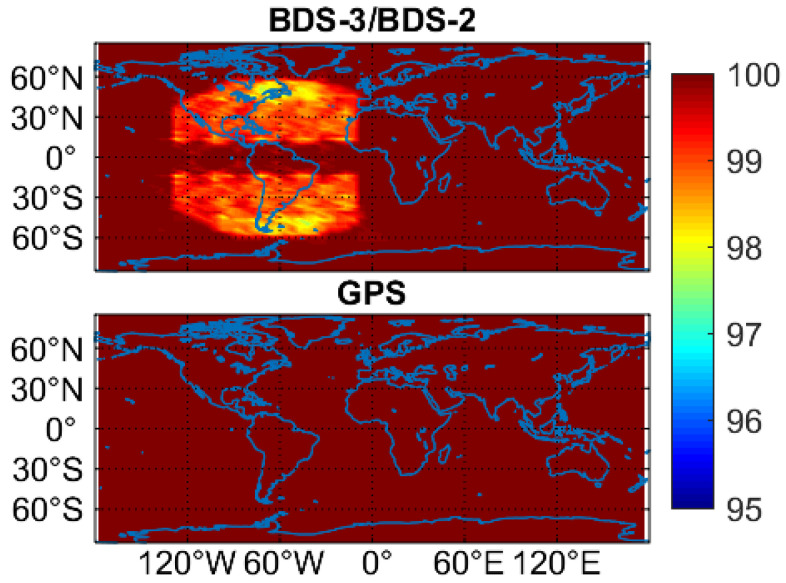
PNT service rate for BDS-3/BDS-2 and GPS on DOY 140–146 of 2020.

Figure 4 exhibits the PNT service rate of GPS and BDS-3/BDS-2 during the analysis period (a week). The service rate refers to the percentage of the time, during which the PNT service is available for use, over the total time. When the PDOP value computed with at least four visible satellites is smaller than 30, the PNT service is considered to be feasible. We can see that both BDS-3/BDS-2 and GPS currently offer a nearly full-time PNT service, except for some American areas where the BDS-3/BDS-2 PNT service rate can decline to 97.6%.

**Figure 5:**
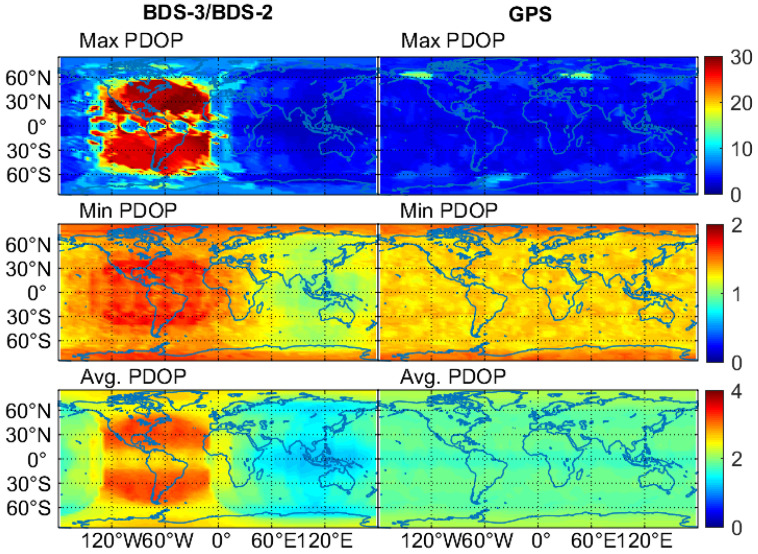
Global maps of the maximum, minimum and average position dilution of precision (PDOP), values for BDS-3/BDS-2 and GPS on DOY 140–146 of 2020.

Figure 5 provides the global maps of the PDOP values. Only the results at the time spans when the PNT service is considered to be feasible are used for the statistics. Corresponding to the satellite visibility, the PDOP values of BDS-3/BDS-2 also demonstrate the viewable regional difference. The minimum value of the maximum and minimum BDS-3/BDS-2 PDOP values is respectively 1.5 and 0.9 in Asia-Pacific regions, and the corresponding maximum value is respectively 30.0 and 1.8 in Americas, resulting in an average PDOP that is 0.4 smaller in Asia-Pacific areas, but 1.0 larger in Americas with respect to that of GPS. The average PDOP values of BDS-3/BDS-2 on a global scale fall between 1.2 and 3.3, while the corresponding fluctuation range is 1.2–1.8 and 1.9–3.3 in the BDS-2 service regions, and the 0°–150° W longitude and 60° S–60° N latitude areas, respectively. As to the GPS, the PDOP values change from 2.1 to 15.8, from 1.2 to 1.6, and from 1.7 to 2.2 for the maximum, minimum and average statistics, respectively.

### 4.3. Quality of BDS-3/BDS-2 Real-Time Precise Products

**Figure 6:**
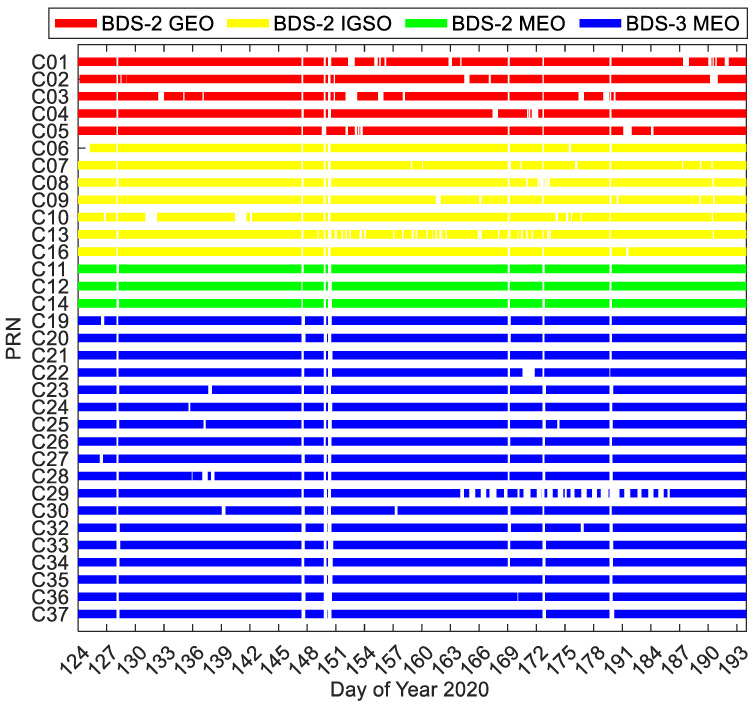
Epoch-wise data availability of BDS-3/BDS-2 real-time precise satellite products.

Figure 6 presents the epoch-wise data availability information of the BDS-3/BDS-2 real-time precise satellite products. The epoch is filled with specific colors for different constellations when the real-time product is available at this epoch, or the corresponding product is unavailable. The data missing can be divided into two types. One is the real-time product missing at the distinct time spans for almost two-thirds of the BDS-3/BDS-2 satellites, and the other one is the real-time product missing at the same time spans for almost all the BDS-3/BDS-2 satellites (e.g., DOY 128 and 147 of 2020). It is seen that the real-time products of IGSO and GEO satellites miss epochs more frequently compared with MEO satellites. The real-time products of several BDS-3 MEO satellites have more data gaps, especially for C29, in comparison with BDS-2 MEO satellites. The operational problems of NTRIP Caster, the scheduled maneuvers, the unstable real-time data streams broadcasted by the ground tracking stations, and other potential factors may result in the unavailability of real-time products.

**Figure 7:**
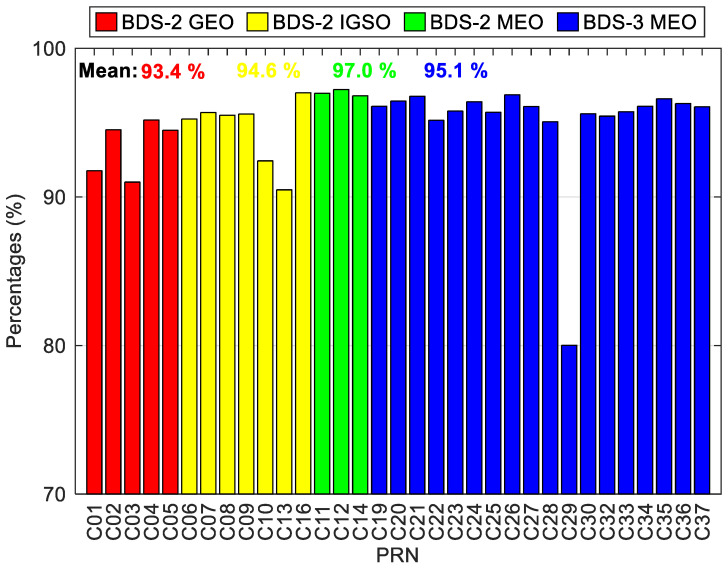
Availability statistics of BDS-3/BDS-2 real-time precise satellite products.

The availability statistic of BDS-3/BDS-2 real-time precise satellite products is shown in Figure 7. The theoretical epoch number is divided by the number of the received epoch-wise real-time corrections to calculate the statistical availability of real-time precise satellite products. There is a satisfied availability for all BDS-3/BDS-2 MEO satellites, which exceeds 95.0%, except for C29, the availability of which is only 80.0%. The availability still exceeds 90.0% for all BDS-2 GEO and IGSO satellites. The average value of availability is computed for each orbit type. The BDS-2 MEO satellites achieve the largest availability of 97.0% on average, followed by the BDS-3 MEO satellites with an average availability of 95.1%. Smaller availability is detected for the BDS-2 GEO and IGSO satellites, which is 93.4% and 94.6% on average, respectively. For comparison, we also calculate the average availability of GPS real-time precise satellite corrections derived from CLK93, and the obtained statistic with a value of 99.3% is found to be larger than the BDS-3/BDS-2 ones.

**Figure 8:**
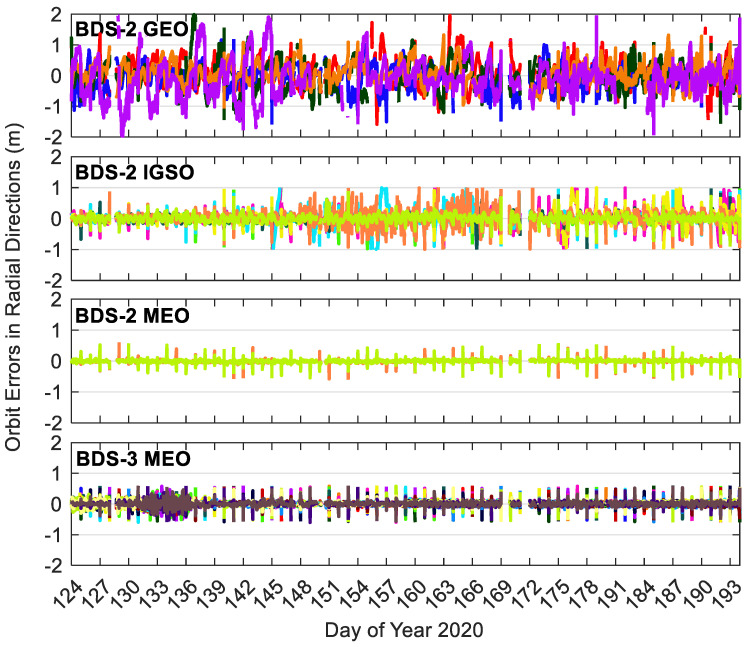
Radial errors of BDS-3/BDS-2 precise satellite orbits in the real-time situation. Different colors in the figure refer to different satellites.

**Figure 9:**
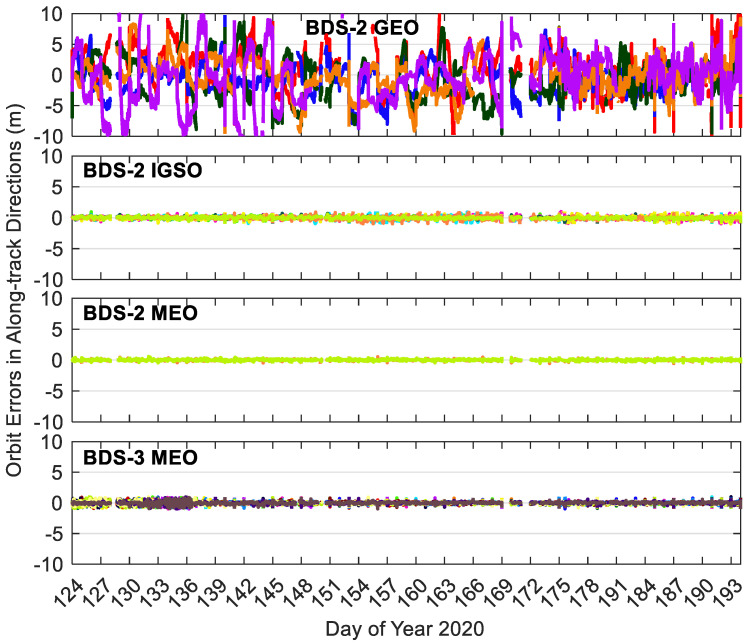
Along-track errors of BDS-3/BDS-2 precise satellite orbits in the real-time situation. Different colors in the figure refer to different satellites.

**Figure 10:**
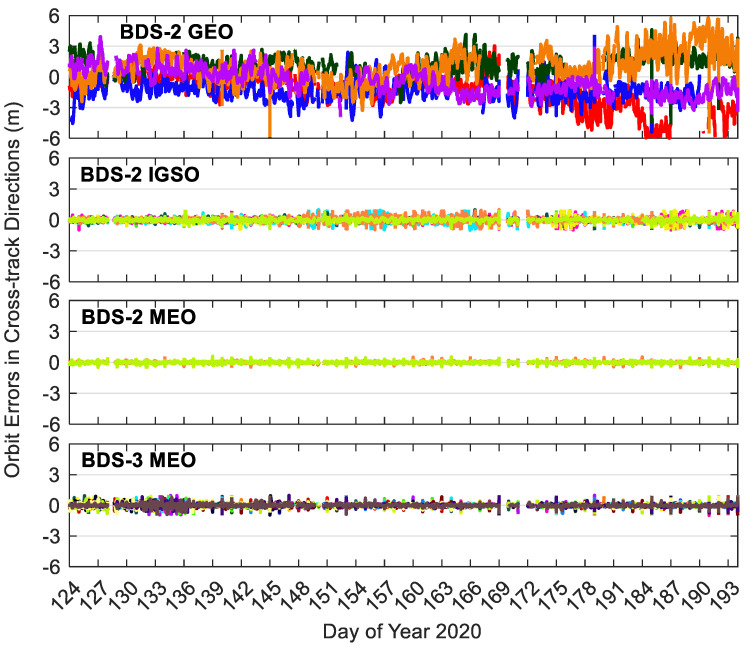
Cross-track errors of BDS-3/BDS-2 precise satellite orbits in the real-time situation. Different colors in the figure refer to different satellites.

**Figure 11:**
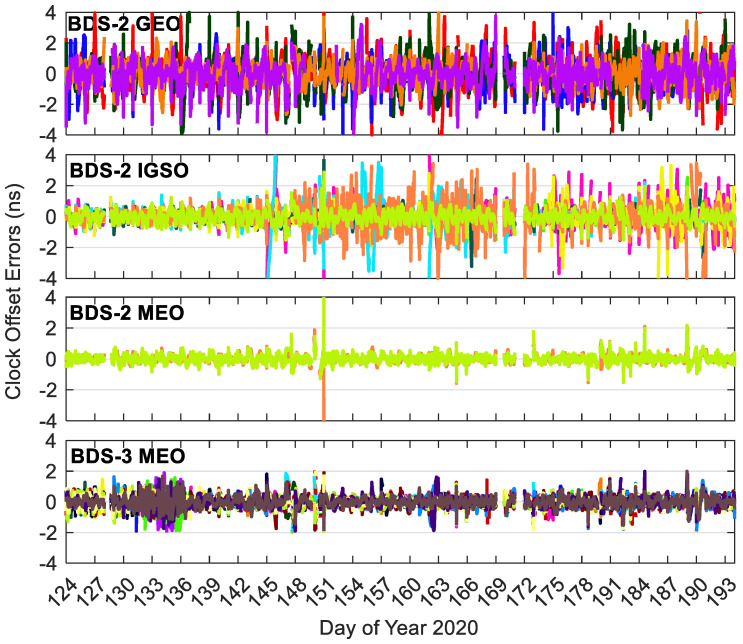
Errors of BDS-3/BDS-2 precise satellite clocks in the real-time situation. Different colors in the figure refer to different satellites.

Figure 8, Figure 9 and Figure 10 show the BDS-3/BDS-2 real-time precise satellite orbit errors in the three directions. Figure 11 presents the corresponding satellite clock errors. Different colors represent different satellites. The final precise satellite products from WHU are taken as references. Both real-time satellite orbit and clock offset errors have frequent spikes, indicating that the stability of BDS-3/BDS-2 real-time precise products should be further enhanced. The along-track and cross-track orbit quality are lower than the radial one. Compared with non-MEO satellites, the error magnitudes of MEO orbit and clock products are smaller, and the qualities of BDS-2 MEO products are comparable with respect to those of BDS-3 MEO satellites. Except for the larger orbit errors on DOY 131–136 of 2020 for BDS-3 MEO satellites as well as the spikes, the orbit errors vary in a similar range for the BDS-3/BDS-2 MEO satellites, which is −0.3 to 0.3, −0.3 to 0.3, and −0.1 to 0.1 m in the cross-track, along-track and radial directions, respectively. The varying range of orbital errors for the BDS-2 IGSO satellites is increased by a factor of 1.5 to 2.5 times in comparison with the MEO satellites. As to the BDS-2 GEO satellites, they achieve the largest noise level of orbit errors, which is −6.0 to 6.0, −10.0 to 10.0, and −1.5 to 1.5 m in the three directions, respectively. For the BDS-3/BDS-2 MEO satellites, the errors of real-time satellite clocks fall between −0.5 and 0.5 ns, whereas the corresponding variation range of BDS-2 GEO and IGSO satellites is expanded four times and twice, respectively.

**Figure 12:**
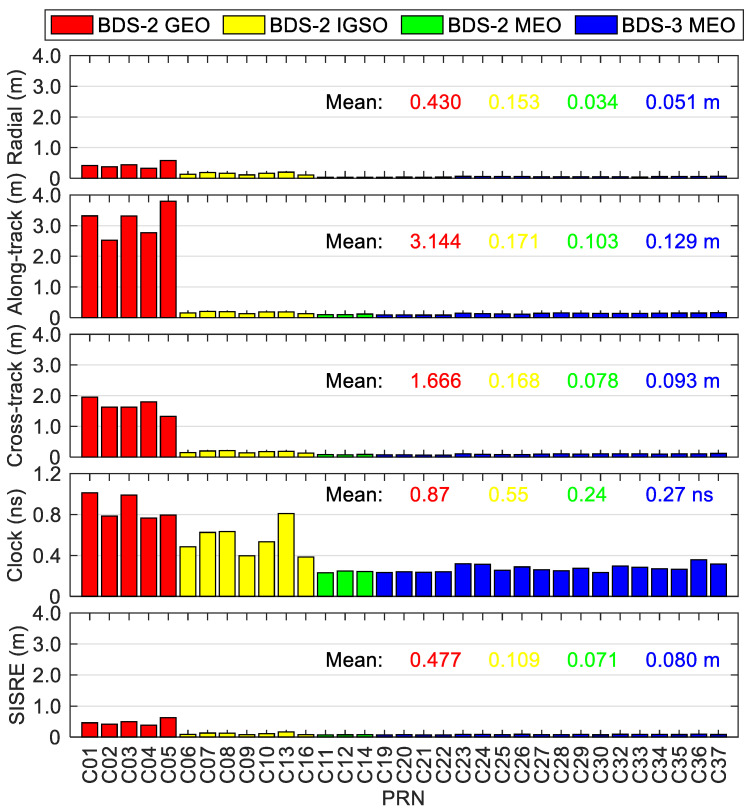
Statistics of errors of BDS-3/BDS-2 real-time precise satellite orbits and clocks.

**Figure 13:**
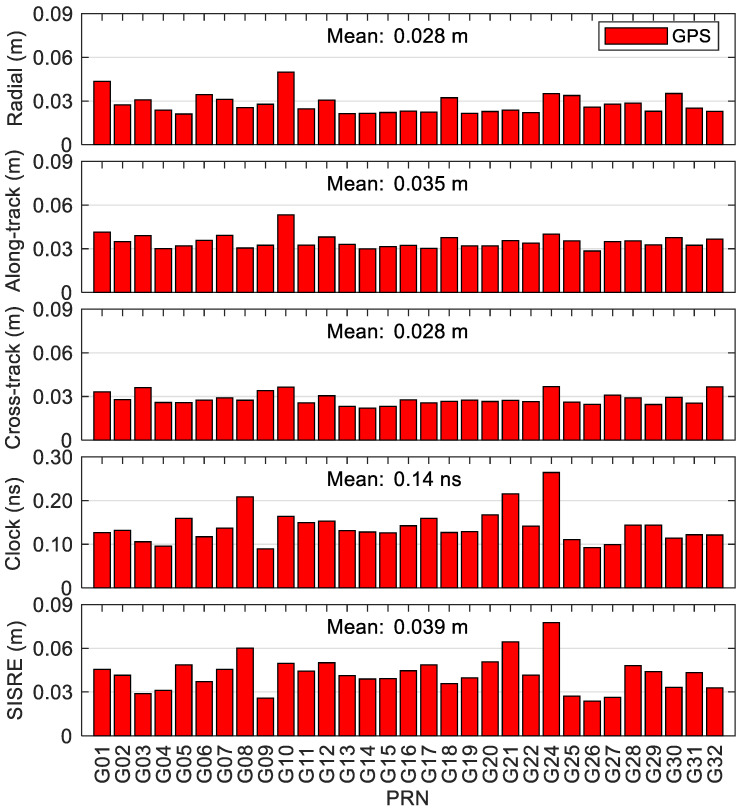
Statistics of errors of GPS real-time precise satellite orbits and clocks.

Figure 12 provides the statistics of errors of BDS-3/BDS-2 precise satellite clock and orbit corrections in the real-time scenario. The satellite-dependent statistical values are at a similar level for the satellites belonging to the same orbit types. It can be seen that the average values of the root mean squares (RMSs) of cross-track/along-track/radial orbit errors over the analysis period (70 days) for the satellites within the same satellite constellations are 1.666/3.144/0.430 m for BDS-2 GEO satellites, and 0.168/0.171/0.153 m for BDS-2 IGSO satellites, which are significantly larger than the corresponding RMSs of MEO satellites of BDS-2 and BDS-3 at the centimeter level except for the along-track direction. For BDS-2 GEO and IGSO satellites, the average RMS clock errors are respectively 0.87 and 0.55 ns, which are nearly two to three times larger than the corresponding RMSs of MEO satellites of BDS-2 and BDS-3. The comprehensive contributions of satellite clock and orbit errors to range measurement are usually expressed by the SISRE [35]. The accuracies of real-time BDS-2 GEO and IGSO precise products are 0.477 and 0.109 m in terms of the mean SISRE, respectively, and those of MEO satellites are similar, i.e., 0.080 m for BDS-3 MEO satellites and 0.071 m for BDS-2 MEO satellites, respectively. Figure 13 shows the statistics of errors of GPS precise satellite products in the real-time situation with respect to IGS final products. The mean cross-track/along-track/radial orbit accuracies of GPS are 0.028/0.035/0.028 m and the mean clock accuracies are 0.14 ns, and the corresponding statistics of MEO satellites of BDS-2 and BDS-3 are 1.2–3.7 times larger than the GPS ones (see Figure 12). It is clear that better stability can be achieved for the GPS real-time precise products, and the mean SISRE is 0.039 m, which is about half of that of MEO satellites of BDS-2 and BDS-3.

### 4.4. Performance of Real-Time BDS-3/BDS-2 PPP

The datasets from 147 stations spanning a week are adopted to evaluate the real-time BDS-3/BDS-2 PPP performance in both kinematic and static scenarios. The BDS-3/BDS-2 post-processed PPP solutions as well as the solutions using GPS observations in both post-processing and real-time modes are also generated for comparison. The final precise satellite products from IGS and WHU are used for GPS PPP and BDS-3/BDS-2 PPP in post-processing mode, respectively. The PPP processing is conducted day-by-day, and the daily position solutions are used for analysis in this section. It should be noted that PPP usually features high position errors at the beginning of the day, and then the position errors gradually decrease. Actually, the change process of position errors from a high level to an acceptable level is not corrected here, and the convergence time is usually taken as an index to describe this. As to the statistic of position accuracies, only the fully converged position solutions after a long processing time are employed.

**Figure 14:**
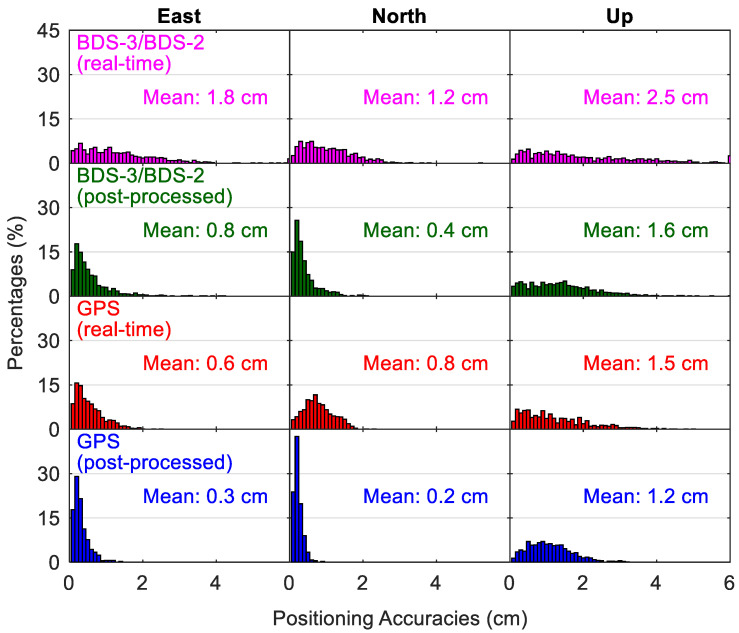
Distribution of positioning accuracies from different static precise point positioning (PPP) solutions.

**Figure 15:**
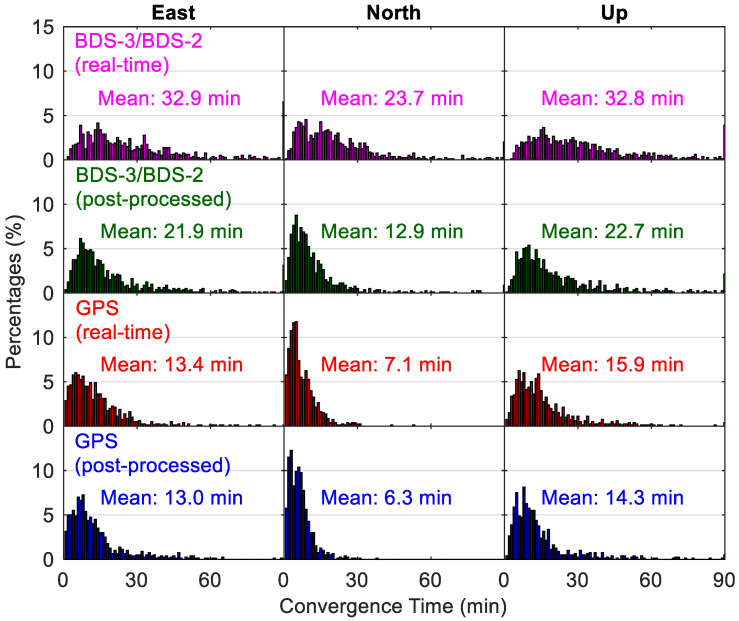
Distribution of convergence time from different static PPP solutions.

Figure 14 and Figure 15 show the distribution of positioning accuracies and convergence time from different static PPP solutions, respectively. The percentage of position solutions with positioning accuracies worse than 6 cm is indicated by the bar near the 6 cm in Figure 14, and it is a similar case for the position solutions with convergence time longer than 90 min in Figure 15. The absolute value of the position error at the last epoch for each 24-h session is taken as the fully converged positioning accuracy of this data set in the static mode. The convergence of positioning at a specific epoch is identified when the position errors are lower than 0.1 m for the next 10 epochs. We take the time spans from the first epoch to the converged epoch as the convergence times. The BDS-3/BDS-2 real-time PPP solutions with static position accuracies better than 2 cm account for 77.7%, 92.0% and 60.6% in the east, north and up directions, respectively, whereas the derived statistics are 62.9%, 77.6% and 58.7% in the three directions for the corresponding position filters with convergence times shorter than 30 min, respectively. The statistics about the convergence time are derived from an accumulation of the percentages for the bars with a convergence time lower than 30 min in Figure 15. The average results over all the employed days and stations are also computed, and they are presented in each panel. We can see that, although the mean east/north/up positioning accuracies using GPS real-time products are higher (i.e., 0.6/0.8/1.5 cm) and the convergence times are also obviously shorter (i.e., 13.4/7.1/15.9 min), the static positioning using BDS-3/BDS-2 real-time products can also converge to the centimeter level within about 30 min, with mean east/north/up position accuracies of 1.8/1.2/2.5 cm and convergence time of 32.9/23.7/32.8 min. The solutions of BDS-3/BDS-2 static positioning have mean east/north/up position accuracies of 0.8/0.4/1.6 cm in the post-processing mode, which are only slightly larger than the values of 0.3/0.2/1.2 cm of GPS post-processed results, but the convergence performance of the BDS-3/BDS-2 one is still significantly worse than the GPS one. The convergence of BDS-3/BDS-2 post-processing solutions consumes dramatically less time than the BDS-3/BDS-2 PPP using real-time products (on average 10.6 min of reduction over the three directions), and the average value is 21.9/12.9/22.7 min. In contrast, those GPS static positioning using real-time products has a similar convergence performance with its post-processing solutions, i.e., 13.0/6.3/14.3 min, which also confirms the high quality of GPS precise satellite products in the real-time situation. As to the accuracy difference between post-processed and real-time solutions in static mode, it is also notable for the BDS-3/BDS-2 PPP (on average 0.9 cm over the three directions), but at a smaller level for the GPS PPP (on average 0.4 cm over the three directions).

**Figure 16:**
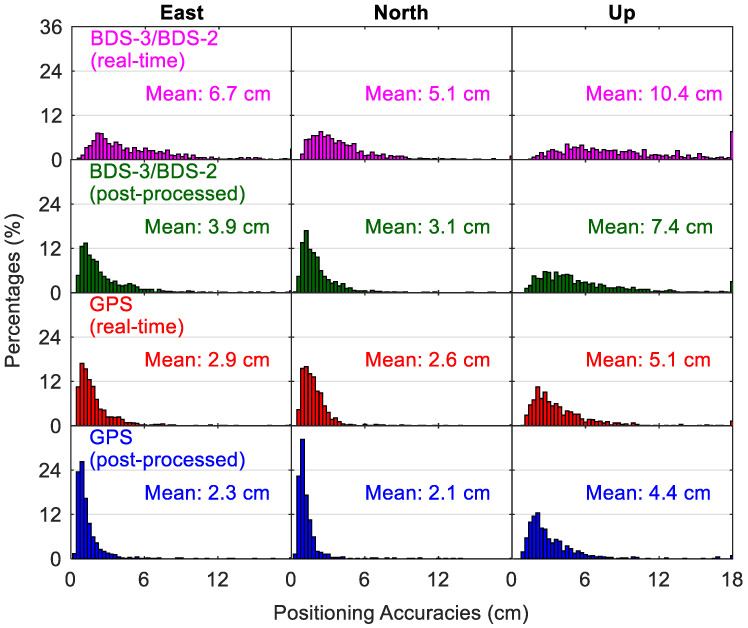
Distribution of positioning accuracies from different kinematic PPP solutions.

**Figure 17:**
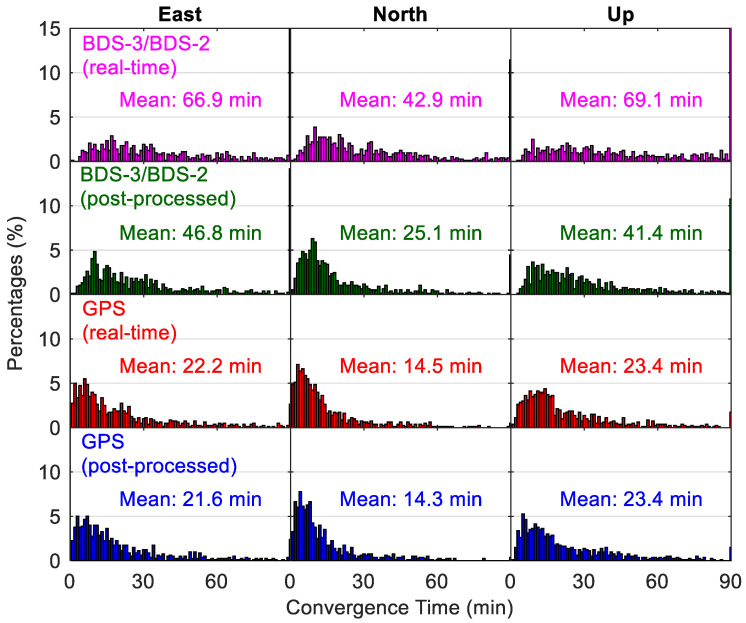
Distribution of convergence time from different kinematic PPP solutions.

Figure 16 shows the kinematic positioning accuracies using different precise products, and Figure 17 compares the corresponding time for convergence. As the kinematic position errors may still fluctuate at a level of several centimeters even after a long processing time, for each 24-h session, the RMS statistics of the positioning errors over the last two hours are computed to indicate the kinematic positioning accuracies. For real-time kinematic PPP with BDS-3/BDS-2 observations, there are 87.1%, 94.7% and 59.8% of solutions with position accuracies better than 9 cm in the east, north and up directions, respectively, whereas the corresponding position filters with convergence times shorter than 60 min account for 64.3%, 80.0% and 59.6% in the three directions, respectively. The mean position accuracies of BDS-3/BDS-2 real-time kinematic PPP are 6.7/5.1 cm in east/north directions, and 10.4 cm for the vertical direction, which are worse than the GPS-derived results with statistics of 2.9/2.6 cm and 5.1 cm respectively. A mean convergence time of 66.9/42.9/69.1 min can be achieved for the BDS-3/BDS-2 real-time kinematic PPP in the east/north/up directions, respectively, which is almost thrice that of GPS results. For the post-processing positioning, the use of BDS-3/BDS-2 final precise products leads to cm-level improvement of mean position accuracies (on average 2.6 cm over the three directions) and about 20-min reduction of convergence time (on average 21.9 min over the three directions) compared to the real-time solutions. Improvements between GPS post-processing solutions and real-time solutions are mm-level in position accuracies and several seconds in convergence time, indicating that the accuracies of GPS precise products in the real-time situation are close to those of GPS final precise products. Compared with the BDS-3/BDS-2 post-processed solutions, the improvements of GPS post-processed solutions in the kinematic mode are 41%/32%/41% and 54%/43%/43% for the position accuracies and convergence time in the east/north/up directions, respectively.

The space weather event can deteriorate the PPP precision, such as magnetic storms [39], with an expansion of the auroral oval [40], solar flares [41], and ionospheric scintillations [42]. The condition of geomagnetic activity during the studied period (DOY 140–146 of 2020 in this section) is also analyzed. During the analysis period, the geomagnetic activity is at a weak level, and the varying range is 0.7 to 1.3, and −7 to −1 nT for the daily mean value of Kp index and Dst index, respectively. Thus, the accuracy differences among different days are not significant for the BDS-3/BDS-2 real-time PPP. It should be noted that the derived positioning performance of BDS-3/BDS-2 real-time PPP in this study could degrade when the geomagnetic activity becomes strong.

## 5. Conclusions

In recent years, there has been a rapid growth in demand for real-time precise applications. With a fully deployed BDS-3 constellation and available real-time precise satellite corrections contained in the real-time stream CLK93 transmitted by CNES, the real-time PPP with BDS-3 observations becomes feasible. Most importantly, the BDS-3/BDS-2 real-time PPP service can provide global coverage. We initially evaluate the global positioning performance of BDS-3/BDS-2 real-time PPP using the datasets from 147 stations in this study. In addition, the BDS-3/BDS-2 post-processed PPP results are also presented. For the purpose of completeness, the availability of BDS-3/BDS-2 constellations in the real-time situation is analyzed in terms of satellite numbers, PNT service rates and PDOP values, and the quality of BDS-3/BDS-2 real-time precise products is assessed. The corresponding results of GPS are also provided for comparative analysis.

The joint use of BDS-3 and BDS-2 observations can offer a nearly full-time PNT service, except for some Americas areas where the BDS-3/BDS-2 PNT service rate can be decreased to 97.6%. The average number of visible BDS-2 and BDS-3 satellites changes from 6 to 19 on a global scale, whereas the corresponding varying range for the average PDOP values is 1.2–3.3. In the BDS-2 service regions (Asia-Pacific regions), the average BDS-3/BDS-2 satellite number is 13–19, while the maximum value for the maximum/minimum satellite number in these regions can be up to 23/17. Regarding the PDOP value, the average value is 1.2–1.8, and the minimum value for the maximum/minimum PDOP value can be as small as 1.5/0.9 in the Asia-Pacific regions. Except for C29, the availability of real-time precise satellite products exceeds 95.0% for all BDS-3 MEO satellites with a mean value of 95.1%. The average availability of real-time products is 97.0%/94.6%/93.4% for the MEO/IGSO/GEO satellites of BDS-2, respectively. Taking the SISRE as an indicator, the accuracies of precise satellite products in the real-time situation are at a similar level for BDS-3 and BDS-2 MEO satellites, which are 0.080 and 0.071 m, respectively. Larger SISRE values can be detected for the other two constellations, i.e., 0.477 m for BDS-2 GEO satellites and 0.109 m for BDS-2 IGSO satellites. In the static mode, the position accuracy of 1.8, 1.2 and 2.5 cm, and convergence time of 32.9, 23.7 and 32.8 min in the east, north and up directions can be achieved for BDS-3/BDS-2 real-time PPP, while the corresponding statistics are 6.7, 5.1 and 10.4 cm, and 66.9, 42.9 and 69.1 min in the three directions in the kinematic mode, respectively. The BDS-3/BDS-2 post-processed PPP improves the position accuracies by 2.6 and 0.9 cm on average, and shortens the convergence time by 21.9 and 10.6 min on average in the kinematic and static modes in comparison to the corresponding real-time PPP results, respectively.

Compared with the BDS-3/BDS-2 results, almost all aspects of GPS results exhibit better performance. The global PNT service of GPS is always available. On a global scale, the average PDOP values change from 1.7 to 2.2, and the varying range is 8.8–10.7 for the average number of GPS visible satellites. The availability of GPS real-time precise satellite products can be up to 99.3%, and the average SISRE is as small as 0.039 m. Significantly better positioning performance can be achieved for the GPS real-time PPP, with a statistic of 0.6/0.8/1.5 cm for static positioning accuracies, of 13.4/7.1/15.9 min for static convergence time, of 2.9/2.6/5.1 cm for kinematic positioning accuracies, and of 22.2/14.5/23.4 min for kinematic convergence time in the east/north/up directions, respectively. Different from BDS-3/BDS-2, due to the high quality of real-time precise products, GPS real-time PPP only has slightly worse positioning performance than GPS post-processed PPP.

## Figures and Tables

**Table 1:** Space segment of BeiDou navigation satellite system (BDS)-3 and BDS-2 as of September 2020.

Generation	Constellation	PRN
BDS-2	GEO	C01–C05/(C18)
	IGSO	C06–C10/C13/C16
	MEO	C11/C12/C14
BDS-3S	IGSO	(C31/C56)
	MEO	(C57/C58/C?)
BDS-3	GEO	(C59–C61)
	IGSO	(C38–C40)
	MEO	C19–C30/C32–C37/(C41–C46)
